# Fabrication and Optimization of 3D-Printed Silica Scaffolds for Neural Precursor Cell Cultivation

**DOI:** 10.3390/jfb14090465

**Published:** 2023-09-09

**Authors:** Georgia Kastrinaki, Eleftheria-Maria Pechlivani, Ioannis Gkekas, Nikolaos Kladovasilakis, Evdokia Gkagkari, Spyros Petrakis, Akrivi Asimakopoulou

**Affiliations:** 1 Chemical Process Engineering Research Institute, Centre for Research and Technology Hellas, 57001 Thessaloniki, Greece; evdokia.gkagkari@certh.gr (E.G.); asimak@certh.gr (A.A.); 2 Information Technologies Institute, Centre for Research and Technology Hellas, 57001 Thessaloniki, Greece; riapechl@iti.gr (E.-M.P.); nikoklad@iti.gr (N.K.); 3 Institute of Applied Biosciences, Centre for Research and Technology Hellas, 57001 Thessaloniki, Greece; gkekasioannis@certh.gr (I.G.); spetrak@certh.gr (S.P.)

**Keywords:** additive manufacturing, neural precursor cells, silica scaffolds, monolithic structure

## Abstract

The latest developments in tissue engineering scaffolds have sparked a growing interest in the creation of controlled 3D cellular structures that emulate the intricate biophysical and biochemical elements found within versatile in vivo microenvironments. The objective of this study was to 3D-print a monolithic silica scaffold specifically designed for the cultivation of neural precursor cells. Initially, a preliminary investigation was conducted to identify the critical parameters pertaining to calcination. This investigation aimed to produce sturdy and uniform scaffolds with a minimal wall-thickness of 0.5 mm in order to mitigate the formation of cracks. Four cubic specimens, with different wall-thicknesses of 0.5, 1, 2, and 4 mm, were 3D-printed and subjected to two distinct calcination profiles. Thermogravimetric analysis was employed to examine the freshly printed material, revealing critical temperatures associated with increased mass loss. Isothermal steps were subsequently introduced to facilitate controlled phase transitions and reduce crack formation even at the minimum wall thickness of 0.5 mm. The optimized structure stability was obtained for the slow calcination profile (160 min) then the fast calcination profile (60 min) for temperatures up to 900 °C. In situ X-ray diffraction analysis was also employed to assess the crystal phases of the silicate based material throughout various temperature profiles up to 1200 °C, while scanning electron microscopy was utilized to observe micro-scale crack formation. Then, ceramic scaffolds were 3D-printed, adopting a hexagonal and spherical channel structures with channel opening of 2 mm, and subsequently calcined using the optimized slow profile. Finally, the scaffolds were evaluated in terms of biocompatibility, cell proliferation, and differentiation using neural precursor cells (NPCs). These experiments indicated proliferation of NPCs (for 13 days) and differentiation into neurons which remained viable (up to 50 days in culture). In parallel, functionality was verified by expression of pre- (SYN1) and post-synaptic (GRIP1) markers, suggesting that 3D-printed scaffolds are a promising system for biotechnological applications using NPCs.

## 1. Introduction

Recent advances in the field of scaffolds for tissue engineering [1,2,3,4,5,6], organoid [7,8,9], and organ-on-a-chip developments [10,11] have raised awareness for controlled 3D cell structures and shapes that mimic the complex biophysical and biochemical cues present in a versatile in vivo microenvironment. The 3D structure can either be synthesized by printing a biocompatible scaffold such as natural polymers (e.g., collagen, fibrin, hyaluronic acid) [12,13], polylactic acid [14,15], polyglycolic acid [16], silk [17], or a ceramic structure such as hydroxyapatite and tricalcium phosphate [18,19] on which cells are deposited either simultaneously with the scaffold material in the former or are cultivated on the scaffold surface at the latter.

Three-dimensional bioprinting technology enabled the precise deposition of diverse cell types [20,21], bioinks [22,23], and biomaterials, culminating in the fabrication of intricate tissue [24] and organ constructs with exceptional cellular-level precision. Despite persistent challenges such as sustaining long-term tissue viability and navigating regulatory considerations [25,26], recent advances in 3D bioprinting exhibit immense potential for the prospect of tailored functional tissue replacements and the acceleration of drug development processes. Considering neural cells [27,28], their cultivation and proliferation after 3D bioprinting remains a challenge, encountering obstacles concerning the viability, functionality, proliferation, and interconnection of neural cells under bioink formulations. The vital requirement of vascularization for supplying nutrients and oxygen to neural tissues further underscores the intricacies of bioprinting. Moreover, the crucial role of synaptic connections among neurons is a major challenge for bioprinting techniques to accurately replicate alignment of neurons and functional synapse formation [27], mirroring the complexity of neuronal networks. Current studies are aiming to develop bioinks that offer appropriate mechanical and biochemical cues to support neural cell growth, differentiation, cell adhesion, migration, and neurite outgrowth, while upholding biocompatibility standards.

On the other hand, oxide ceramic structures such as silica [29,30,31], alumina [32,33], and ceria [34,35] synthesized by bottom-up and top-down techniques offer an advantageous scaffold for cell culture, since the porosity and crystallinity of these materials can be tailor controlled providing both adhesion characteristics for uniform cell distribution and improved nutrient exchange while the surface cell deposition allows access for live imaging and in vitro characterization that simulates cells in an organ-like environment. Ceramic scaffolds, apart from the conventional fabrication techniques (e.g., sol-gel, foaming, extrusion, machining, etc., [36,37,38]) are often produced using state-of-the-art additive manufacturing techniques, such as 3D printing, that provide high-resolution control over their architecture and complexity [4,39]. Ceramic scaffolds which are 3D-printed offer an added value platform with improved structural and functional properties for 3D cell culture, adhesion, growth, and tissue engineering. Such structures can contribute to organoid applications and the development of biosensors [40] consisting of human neurons, offering nutrient availability, oxygen levels, and mechanical properties, which are important for successful organoid growth. The important physicochemical parameters that promote cell adhesion and proliferation conditions are the crystallinity of the material, hydrophilicity, material homogeneity, and avoiding cracks. Silica scaffolds have been produced using 3D printing technology with a Methacrylate/Silica composite [41]. In addition, a silicate scaffold was fabricated utilizing silicate fibers [42]. However, no studies have been conducted so far regarding the investigation of a 3D-printed pristine silica ceramic scaffold.

**Figure 1:**
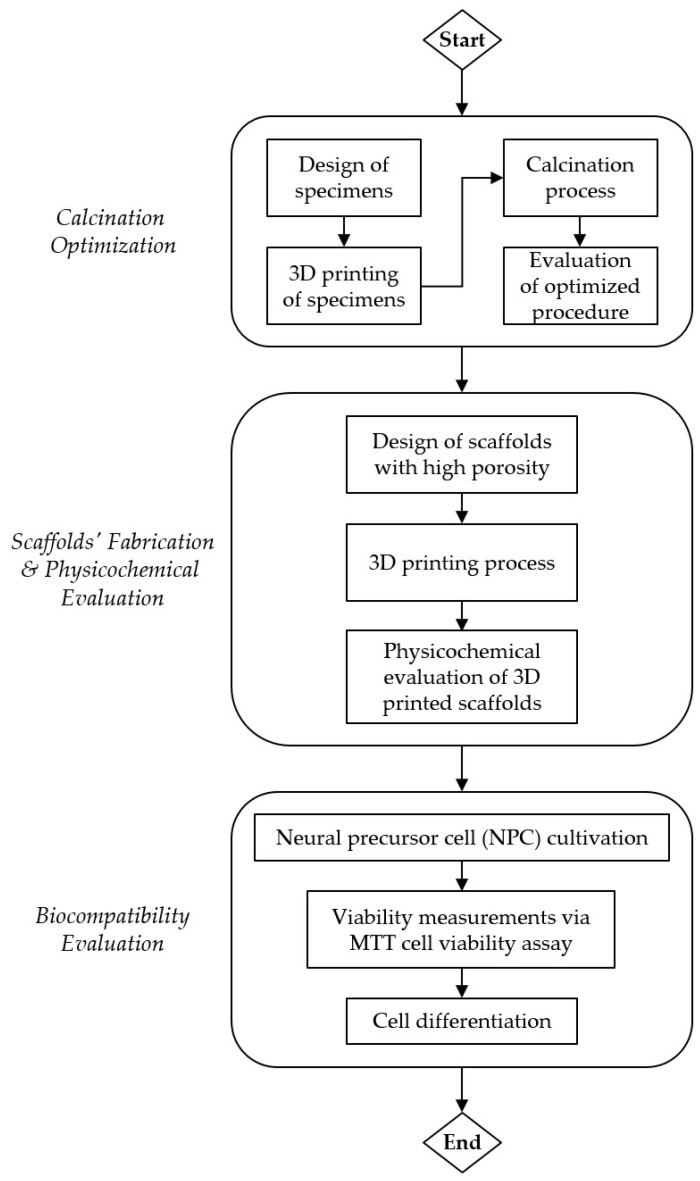
Flowchart of the current study.

The aim of the current work is to manufacture a 3D-printed monolithic silica scaffold for neural precursor cell (NPC) cultivation that will allow a bottom-up cultivation of neural precursor cells as single points of growth allowing tridimensional evolution of the cells inside the channels of the porous ceramic silica scaffold. Figure 1 presents the flowchart of the current study. In detail, a primary study on the critical calcination parameters was implemented in order to synthesize robust and homogeneous scaffolds with minimum wall thickness, eliminating crack formation. Four cubic scaffolds with wall thickness from 0.5 mm to 4 mm were printed and calcined at two different calcination profiles (Fast and Slow). Thermogravimetric (TGA) analysis was implemented on the printed (fresh) material initially exhibiting at the Fast profile displaying the critical temperatures with increased mass loss; at the current values isothermal steps were added to allow controlled phase change and a reduction in crack formation. In situ X-ray diffraction (XRD) study exhibited the crystal phases of the material at different temperature profiles while scanning electron microscopy (SEM) depicted crack formation at the micro-scale. The ceramic scaffolds were manufactured by 3D printing at hexagonal and spherical channel structures, calcined at the optimized profile, and applied for neural NPC cultivation.

## 2. Materials and Methods

### 2.1. Design and Additive Manufacturing

The 3D-printed calcination specimens were initially designed in SolidWorks^®^ Professional 2022 SP 2.0 software and were constructed by a composite ceramic resin. The printed structure was calcined, resin removal took place initially, and at higher temperature sintering of the ceramic forms the final structure. More specifically, the developed designs were additively manufactured via the stereolithography technique (SLA) utilizing a Formlabs Form-2 3D printer. The employed construction material was the composite ceramic resin of the Form X ceramic resin. This composite material consisted of a photopolymeric matrix with silica (SiO_2_) particles as reinforcement. During the calcination process, the substantial mass of material (polymer’s mass) was burnt away resulting in an extensive shrinkage effect. For this reason, the 3D models were scaled up 1.123 times to achieve the desired final dimensions for the specimens. Furthermore, the minimum layer height of 50 μm was selected in order to produce specimens with high accuracy and quality. Regarding the build orientation of the specimens, the employed orientation of the specimens was the XZY, according to the ASTM standards [43], in order to ensure the minimum support and the maximum accuracy. The printing material was ceramic resin (Form X, Formlabs).

### 2.2. Physicochemical Characterizations

**Figure 2:**
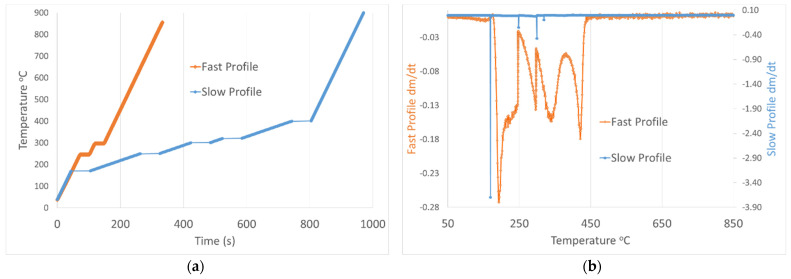
(**a**) Fast and Slow temperature profile. (**b**) Mass loss derivative as obtained from TGA for Fast and Slow temperature profile.

The HR (high resolution) mapping image was calculated by a micro-Raman Renishaw, Qontor, Gloucestershire, UK in Via Instrument coupled with a solid state 532 cm^−1^ laser and ×10 Leica lens. The analyzed area was 255 × 192 μm and the step resolution was 1 μm. We collected 51,400 spectra which were analyzed by the Wire 5.3 software for the digital construction of the HR mapping image. The color map was created with respect to the intensity of the spectrum at the characteristic silica peak at 400 cm^−1^. The SEM images were taken by a JEOL IT500, Tokyo, Japan instrument at the high vacuum mode and at 20 kV operating voltage. The thermogravimetric analysis was performed on 3D-printed material, grinded by a pestle into powder with a thermogravimetric analyzer (Perkin Elmer Pyris-6, Shelton, US, TGA) and heated under 20% O_2_ in N_2_ at a Slow and Fast temperature profile as explained in Figure 2. The in situ XRD was a D8 Advance from Bruker equipped with Cu Ka radiation source from 5–80 2θ angle with 0.04 step and the high temperature oven was a HTK 1200N from Anton Paar. The wetting ability of the ceramic material was measured by the water contact angle method in an in-house contact angle measurement instrument based on a high-resolution sensor camera (Nikon D5600, Tokyo, Japan, AF-P NIKKOR 18–55 mm 1:3.5–5.6 G) according to the arrangement proposed in [32]. Micro-droplets (2.5 μL) of a double distilled water drop were added manually via a variable volume 0–20 µL Eppendorf Research micropipette.

### 2.3. Wettability Measurements

The wetting ability of the ceramic material was measured by the water contact angle method in an in-house contact angle measurement instrument based on a high-resolution sensor camera (Nikon D5600, Tokyo, Japan, AF-P NIKKOR 18–55 mm 1:3.5–5.6 G) according to the arrangement proposed in [32]. Micro-droplets (2.5 μL) of a double distilled water drop were added manually via a variable volume 0–20 µL Eppendorf Research micropipette.

### 2.4. NPC Cultivation and Differentiation

NPCs were obtained from healthy human induced pluripotent stem cells (iPSCs) (ethical approval: IRB code #EA2/131/13). Human NPCs [44] were cultured in expansion medium composed of DMEM/F12:Neurocult (1:1) supplemented with 0.5X N2 (Gibco, Thermofisher Scientific, Waltham, MA, USA), 0.5X B27 (without vitamin A, Gibco, Thermofisher), 3 μM CHIR99021 (Sigma-Aldrich), 0.5 μM purmorphamine (Sigma-Aldrich, St. Louis, MO, USA), 2 mM L-glutamine (Biowest, Nuaille, France), 150 μM ascorbic acid (Sigma-Aldrich), and 1% penicillin/streptomycin (Biowest) in a humidified incubator (37 °C, 5% CO_2_, 20% O_2_). Matrigel-coated ceramic scaffolds were loaded with NPCs (0.5 × 10^6^ cells per scaffold). Undirected differentiation was performed by culturing in an expansion medium lacking ascorbic acid, CHIR99021, and purmorphamine. Immersion in cell suspension involved incubation for 1 h in 300 μL cell suspension (0.5 × 10^6^ smNPCs) at 600 rpm shaking.

### 2.5. Scanning Electron Microscopy

NPCs grown on ceramic scaffolds were fixed with 4% glutaraldehyde for 30 min before being dehydrated in increasing concentrations of ethanol. Samples were air dried, mounted on SEM stubs using a two-sided adhesive film, and covered by a thin gold coating. Microscopic observation was carried out using a JSM-6300 JEOL SEM instrument (JEOL Ltd., Tokyo, Japan) operating at an accelerating voltage of 20 kV.

### 2.6. MTT Cell Viability Assay

For the estimation of cell viability, NPC-laden ceramic scaffolds were incubated with 0.5 μg/mL MTT (Applichem, Darmstadt, Germany) for 4 h. The medium was removed, and formazan crystals were formed by cells dissolved in 300 μL DMSO (Applichem) by shaking at 37 °C for 1 h. The absorbance at 570 nm and 630 nm was measured in a SPARK plate reader (Tecan, Männedorf, Switzerland).

### 2.7. RNA Isolation and Reverse Transcription-Quantitative Polymerase Chain Reaction (RT-qPCR)

Total RNA was purified from control or differentiated NPCs grown on ceramic scaffolds. The cells were detached using a 3:1 mixture of Accutase/0.05% Trypsin. The resulting suspension was centrifuged at 300 × *g* for 6 min at room temperature, the supernatant was discarded, and the cell pellet was utilized for RNA isolation. Total RNA was extracted using TRizol (Sigma-Aldrich). RT-qPCR was conducted using the Luna Universal One-Step RT-qPCR Kit (New England Biolabs, Ipswich, MA, USA) according to the manufacturer’s instructions in a Rotor-Gene 6000 (Qiagen, Venlo, The Netherlands) operating system. The primer sequences have been previously described [32]. The correct size of amplified RT-qPCR products was verified by electrophoresis in a 2% agarose gel. The relative mRNA expression was calculated using the 2^−ΔCt^ method and normalized to the housekeeping GAPDH gene.

### 2.8. Statistics

Statistical analysis was performed using the GraphPad Prism software, Version 9 (San Diego, CA, USA). All experiments were performed in triplicate and the results are shown as mean ± SD. Statistical significance was calculated using the Student’s *t*-test.

## 3. Results

### 3.1. Calcination Profile Optimization for Ceramic Adhesion

The recommended wall thickness of the printed parts from the Form X ceramic resin specifications is between 2 and 10 mm; for bioceramic scaffold development the wall thickness should be adjusted to lower values to allow neuronic connections with adjacent cells and formation of a 3D neural network. Additionally, a dense homogeneous scaffold morphology lacking cracks is important for cell adhesion; a smooth surface with a medium wettability factor will promote matrigel absorption, which is the nutrition agent that cells are initially attached to at the stem cell stage.

A physicochemical parametric analysis was performed on the printed material in order to validate and potentially control the mechanisms of polymer mass loss, silica sintering and crystallization during the calcination process, optimize structure stability and solid ceramic formation, and avoid cracking at the macro and micro-scale. The manufacturer calcination profile and respective mass loss with temperature by TGA are exhibited by the orange line (Fast Profile) in Figure 2a,b, respectively, consisting of two ramps and burnout steps at 240 °C and 300 °C and a sintering step at 1271 °C (Supplementary Figure S1), indicating in Figure 2b that the derivative of the mass loss does not reach a minimum at any point and is exhibiting peaks with high FWHM vs. temperature, an outcome that can lead to uncontrolled phase change, mass loss, and cracking formation.

In order to provide more controlled conditions of polymer removal, additional isothermal steps were added at the temperatures of 170 °C, 250 °C, 300 °C, 320 °C, and 400 °C; these temperatures exhibited maximum mass loss in the Fast profile. The final temperature program determines the Slow profile (blue line) in Figure 2a, providing additional time at constant critical temperatures that lead to a more controlled mass loss, contributing to a more stable structure formation. Figure 2b shows that the additional isothermal steps had a near-zero mass loss derivate at most points, an outcome that can contribute to controlled phase change and structure stability.

**Figure 3:**
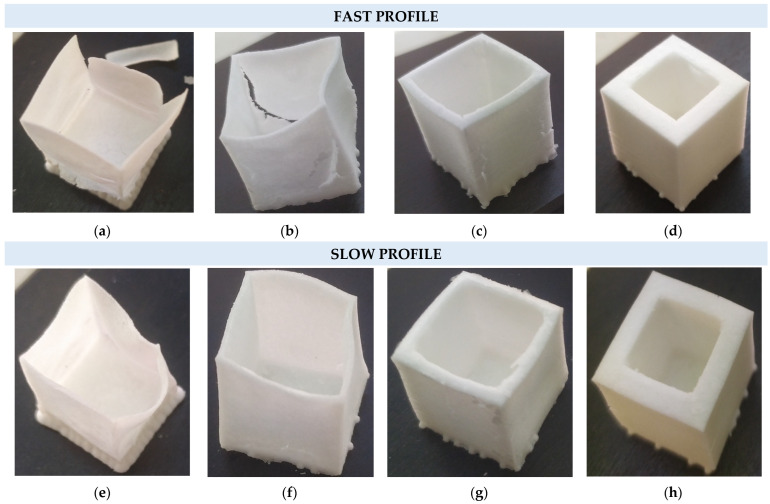
Fast profile of: (**a**) 0.5 mm, (**b**) 1 mm, (**c**) 2 mm, (**d**) 4 mm and Slow profile (**e**) 0.5 mm, (**f**) 1 mm, (**g**) 2 mm, and (**h**) 4 mm.

A series of bulk cubic (2 cm) structures with wall thicknesses of 0.5 mm, 1 mm, 2 mm, and 4 mm were subjected to both temperature profiles, as exhibited in Table 1, in order to macroscopically evaluate the effects of the calcination effect and provide an evaluation of temperature profile optimization. Figure 3a–d shows that for the Fast profile, the only structure that remains without obvious macroscopic cracking or wall collapse is that of 4 mm wall thickness; in the other three cases, the walls have substantial cracks and deformation, probably due to uncontrolled phase change of the polymeric matrix with the temperature increase. The cubic structures being subjected to the Slow profile in Figure 3e–h do not exhibit any cracks, with a partial exception the structure of the thinner wall thickness of 0.5 mm exhibiting a partial deformation due to structure sintering.

**Figure 4:**
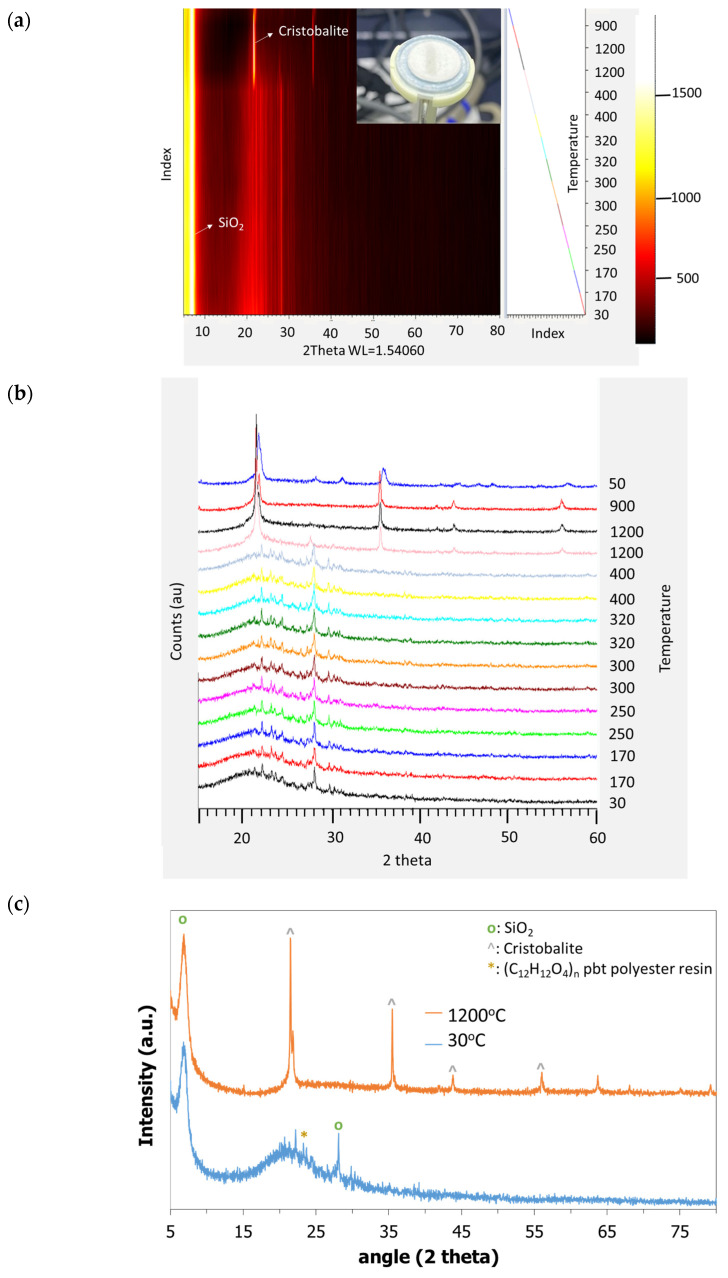
(**a**) Two-dimensional diagram of the respective material intensity XRD peaks with temperature and powder sample placed at the in situ oven holder; (**b**) XRD diagrams at the different temperatures; (**c**) comparison of the diagram at 30 °C and 1200 °C temperatures exhibiting the SiO_2_, cristobalite and PBT peaks.

**Figure 5:**
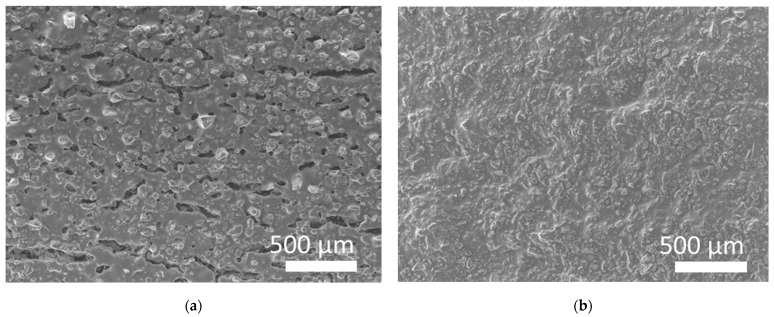
SEM images of the 3D-printed structure with 0.5 wall thickness calcined by the (**a**) Fast profile and (**b**) Slow profile.

Table S1 in the supplementary material shows the dimensions of the uncalcined and calcined samples in the Fast and Slow profiles showing similar reduction rates for both profiles at the macroscale. The as-printed material (uncalcined) was grinded by pestle into powder and was studied by in situ XRD in order to evaluate any potential phase changes and silica crystallization effect with temperature. Figure 4a portrays the powder in the in situ oven holder and the 2D diagram of the material intensity XRD peaks with temperature, Figure 4b exhibits the sequential XRD diagrams at different temperatures and Figure 4c compares the fresh and calcined samples at temperatures of 30 °C and 1200 °C, respectively. The material at room temperature exhibits a high peak at 7 2theta, which is attributed to silicon oxide and a peak at 23.5 2theta which is attributed to (C_12_H_12_O_4_)n poly(butylene terephthalate)-PBT. In temperatures higher than 450 °C, the PBT is eliminated and at 1200 °C the cristobalite phase starts to appear. At 1200 °C the cristobalite exhibits a peak at 21.3 2theta, while at the end of the sintering phase, the cristobalite phase remains at 21.8 with additional peaks at 28, 31, and 36 2theta. In situ XRD shows that at temperatures up to 450 °C, PBT is present but probably gradually oxidized, and thus the additional isothermal steps of the Slow profile have contributed to controlled PBT removal providing sufficient time and space for the remaining structure to form a solid dense structure. The microscopic morphology of the calcined structures of 0.5 mm was characterized by SEM, exhibiting cracks in the case of the Fast profile calcination in Figure 5a, while in the Slow profile case in Figure 5b the wall exhibits a solid dense morphology, which is consistent with the macroscopic view of the sample. A comprehensive study of the crystal phase change and surface area of the ceramic resin material in powder form (by grinding the printed materials) has been implemented in a recent study [45] showing a mesoporosity of 3 and 50 nm and surface area of 6 m^2^/g.

### 3.2. Wettability Measurements

**Figure 6:**
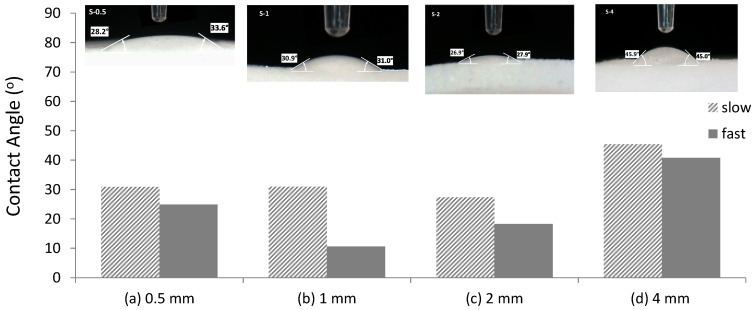
Contact angle values obtained from 2.5 ul drops, highlighting the effect of Slow and Fast calcination. Inserts are optical images showing contact angle values for 2.5 uL of dDI water.

All 3D-printed silica samples established a dynamic wettability behavior, i.e., strongly varied with time due to changes in the surface tension of the water and/or the surface energy of the silica substrate, with rapid full water absorbance (i.e., within the first 2–10 s) when dry samples were used. In order to perform reproducible dynamic water contact angle (WCA) measurements, the prior samples’ immersion in double distilled (DD) water was employed as it modestly affected the solid surface energy and offered a slight delay before the DD water microdroplet of 2.5 μL volume was fully absorbed. The advancing WCA was recorded by the camera and pictures were later extracted by replaying the recorded videos at low speed (i.e., 0.25 of initial speed). Dynamic WCA was measured within a very short period of time (no more than 1 s) of water contact with silica structures calcined under both (i.e., Slow and Fast) heating profiles of (a) 0.5 mm, (b) 1 mm, (c) 2 mm, and (d) 4 mm wall thickness and are shown in Figure 6.

The values of contact angle varied from 27° to 46° and from 25° to 41° for Slow and Fast profiles, respectively. Although the observation of differences in wettability pattern with varying wall thickness is expected due to the variations of surface tension of the water and the surface energy of each silica substrate, the slightly decreased WCA observed for Fast profile can be attributed to the micro-cracks observed also in the SEM images of Figure 5a, which accelerate water absorption on the ceramic structure. Meanwhile in the case of the Slow calcination profile, the WCA has a similar mean value of 30° for the S-0.5, S-1 and S-2, a value that increases to 46° for the S-4 sample, verifying that the Slow profile forms a dense homogeneous structure, a phenomenon that is slightly enhanced for the higher 4 mm wall thickness.

### 3.3. Design and Manufacturing of the 3D-Printed Scaffolds

**Figure 7:**
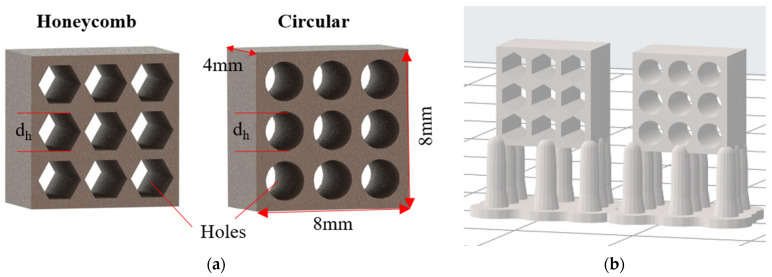
(**a**) Indicative images of the scaffolds 3D designs. (**b**) Indicative image of the orientation of the specimens.

In the current study, ceramic scaffold specimens were designed with orthogonal configuration and external dimensions of 8 mm × 8 mm × 4 mm, as depicted in Figure 7. Hexagonal (honeycomb) and circular channels with the same wall thickness of 0.5 mm were added to the structure in order to attain a 3D network to the scaffold volume domain. Table 2 lists the different types of designed scaffolds with the corresponding channel type, diameter, and porosity (*p = 1 − V_structure_/V_bounding box_*) as determined by the software. In addition, Figure 7a illustrates the design process with linear arrays in order to model the channel pattern. The exact placement of the specimens on the build platform is portrayed in Figure 7b, which was derived from PreForm™ 3.3.3 slicing software. Furthermore, in Table 3, the indicative values of the main properties for the 3D-printed parts made of pure Silica (after the firing process) are listed, according to the manufacturer’s datasheet, coupled with the basic features of the employed SLA 3D-printing process. In addition, in Table 3, the main properties of the 3D-printed part made of pure silica (after the firing process) are listed coupled with basic features of the SLA 3D printing process.

**Figure 8:**
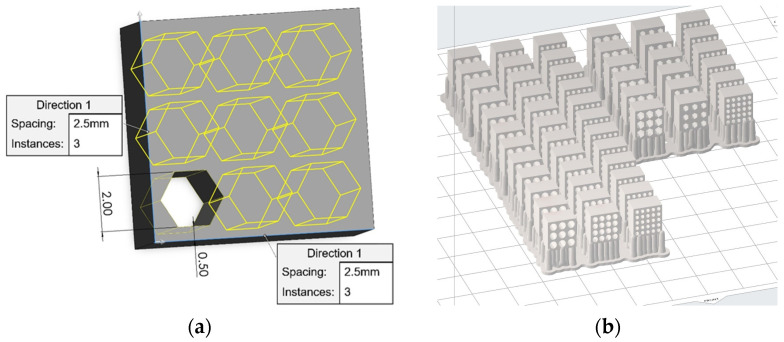

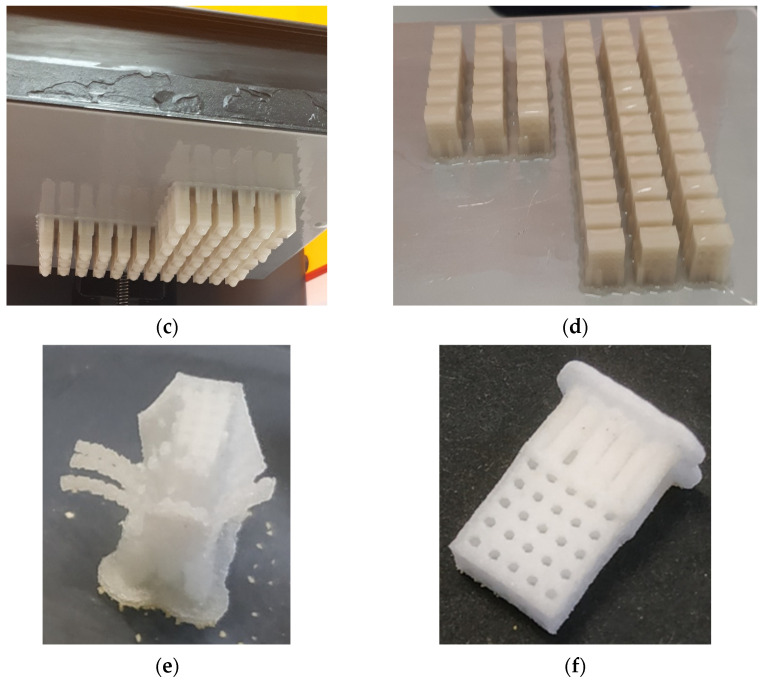
(**a**) Three-dimensional model image from design software, (**b**) image of the specimens in the slicer software, (**c**) image of the 3D-printed specimens during the 3D printing process, (**d**) image of the 3D-printed specimens on the build platform; 3D-printed scaffold calcined at the (**e**) Fast and (**f**) Slow profile.

Finally, Figure 8 presents the images from all steps of the procedure for the production of the 3D-printed silica scaffolds. More specifically, Figure 8a shows the design process and Figure 8b shows the nesting of the scaffolds on the build platform of the SLA 3D printer through the slicer software. Figure 8c presents an indicative image during the 3D printing process and Figure 8d portrays the print-out specimens on the building platform after the 3D printing process. The 3D-printed scaffolds were calcined by both the Fast and Slow profiles and the respective images are depicted in Figure 8e,f, respectively, indicating the effect of structure collapse for the Fast the profile and a denser solid structure for the Slow profile.

### 3.4. Characterization of 3D Calcined Scaffolds

**Figure 9:**
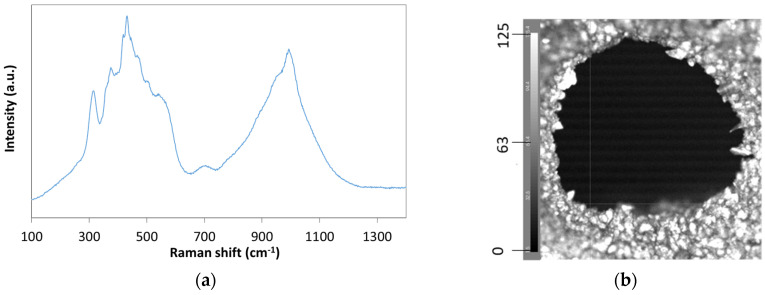

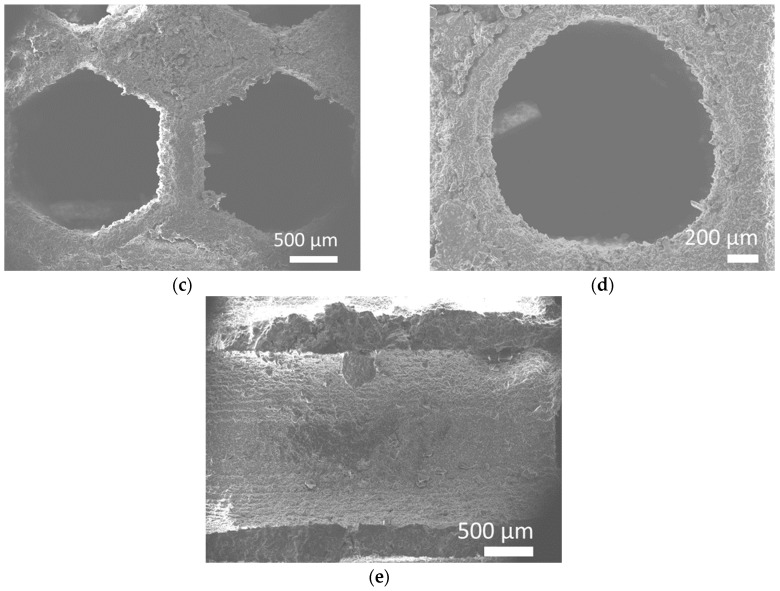
(**a**) Raman spectrum of SiO_2_ scaffold, (**b**) Raman chemical image of a 3D-printed SiO_2_ scaffold with the color gradient indicating the intensity of the 400 cm^−1^ peak; SEM images of the calcined scaffold indicating (**c**) the hexahedral channel, (**d**) the spherical channel, and (**e**) the inner channel surface.

The calcined samples were characterized by Raman analysis to indicate the chemical structure of the scaffold, Figure 9a depicts the characteristic spectrum, where the broadband between 200 and 600 cm^–1^ is associated with the bending modes of SiO_4_ tetrahedral. The broad band between 900 and 1300 cm^–1^ is associated with stretching modes of SiO_4_ tetrahedral and Figure 9b shows the chemical image of the scaffold, with white color indicating the intensity peak of the spectrum at the respective point at 400 cm^−1^. The SEM images of the scaffolds in the rest of Figure 9 depict the hexagonal (Figure 9c) and spherical (Figure 9d) channel structure, while the in-channel morphology depicted in Figure 9e, shows the dense morphology of the ceramic structure at the microscale.

### 3.5. Biocompatibility of 3D-Printed Ceramic Scaffolds with Human NPCs

The compatibility of the printed ceramic scaffolds was assessed with human NPCs. The cells were seeded on scaffolds with a wall thickness of 0.5 mm and various channel diameters (0.5–2 mm) using two methods, either by direct loading or by immersion in NPC suspension medium and gentle shaking at 600 rpm for 1 h at 37 °C. Their viability was measured at various time points post-seeding using MTT, a metabolic assay that highlights cells by the deposition of formazan crystals with a dark blue color. We observed a higher seeding capacity in scaffolds with a 2 mm diameter; furthermore, seeding by immersion in a cell suspension resulted in a more homogeneous distribution of cells within the channels of the scaffold compared to direct cell loading. In both cases, NPCs efficiently populated the scaffold, as indicated by the dark blue color after incubation with MTT and deposition of the formazan crystals by viable cells (Figure S1).

**Figure 10:**
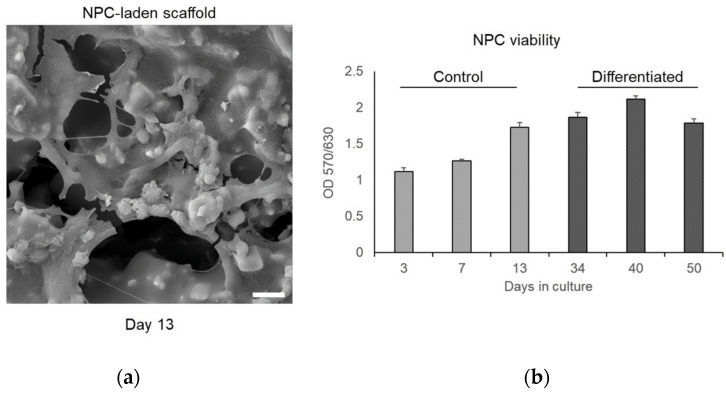
Biocompatibility of 3D-printed ceramic scaffolds with human NPCs: (**a**) indicative SEM image of an NPC-laden scaffold (scale bar = 10 μm); (**b**) viability of control (undifferentiated) and spontaneously differentiated NPCs grown on 3D scaffolds.

The morphology of NPCs cultured on 3D-printed scaffolds was observed by SEM. The cells formed a layer on the surface of the round channel and exhibited outgrowths, a characteristic of NPCs grown in 2D (Figure 10a). The proliferation of NPCs was measured by MTT. The cells almost doubled in number within 13 days of culture (Figure 10b). We have previously shown that human NPCs cultured on 2D alumina substrates efficiently differentiate into neuronal populations and form a functional neuronal network [32]. Therefore, NPCs grown on 3D-printed ceramic scaffolds were spontaneously differentiated for 21 days (day 34 post-seeding) as well. The neuronal cells were maintained in culture up to 50 days post-seeding without any significant decrease in their viability (Figure 10b).

**Figure 11:**
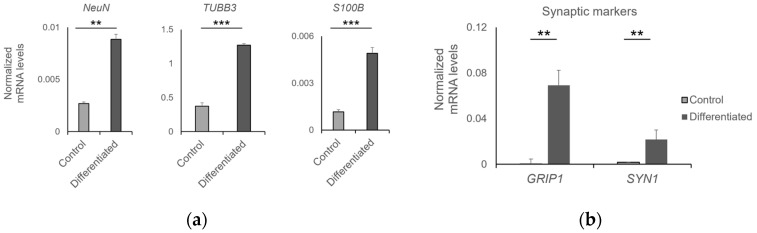
Characterization of neuronal cells grown on 3D-printed ceramic scaffolds. Expression levels of (**a**) neuronal markers NeuN, TUBB3, S100B and (**b**) synaptic markers GRIP1 and SYN1 in control and differentiated NPCs (** *p*-value < 0.01, *** *p*-value < 0.001).

The efficiency of differentiation was determined by RT-qPCR. Differentiated NPCs expressed high levels of NeuN and TUBB3, two markers of mature neurons and S100B, a glial marker, compared to undifferentiated control cells (Figure 11a). This result suggests that the NPC-laden scaffolds contained mixed populations of neurons and glial cells. The functionality of neurons was indirectly assessed by the expression of pre- and post-synaptic markers. Indeed, the differentiated NPCs expressed significantly higher levels of the pre- and post-synaptic markers SYN1 and GRIP1, respectively, suggesting that they may efficiently transduce neuronal signals (Figure 11b).

## 4. Conclusions

Additive manufacturing provides a robust technology to manufacture ceramic scaffolds for cell adhesion and proliferation. In the current work, we demonstrated a parametric study of the calcination profile for the optimization of the micro and macro structure of the scaffold in order to avoid crack formation and attain wall thickness down to 0.5 mm. A Slow calcination profile was proposed, having additional isothermal steps at high mass loss temperatures in contrast to the fast calcination profile of the manufacturer; the Slow profile showed better stability at cubic ceramic structures with wall thickness from 0.5 mm to 4 mm. Orthogonal ceramic structures with hexagonal and cylindrical channels were manufactured by an SLA 3D printer at channels diameters of 1, 1.5, and 2 mm and wall thickness of 0.5 mm, and were calcined by the Slow profile. Both cylindrical and hexagonal channel calcined scaffolds were infused with human NPCs, with cylindrical channels demonstrating a higher proliferation rate for cell culture as assessed by the MTT assay. These scaffolds were subsequently chosen for further evaluation in cell-based bioassays encompassing assessments of viability, proliferation, and differentiation potential. The proliferation rate was constant for 13 days, while differentiated cells remained viable for 50 days; neuronal functionality was confirmed through the expression of both pre-synaptic (SYN1) and post-synaptic (GRIP1) markers. The spatial distribution of proliferated cells, as indicated by the MTT assay, exhibited a preference for the interior of the 2 mm channels. Additionally, the scanning electron microscopy (SEM) images of cells within the channel cross-section revealed interconnections between cells in three dimensions, effectively covering the ceramic surface. The observed cellular proliferation on the scaffold’s surface, coupled with the formation of synaptic connections, holds promise for biotechnological applications of NPC-laden 3D-printed scaffolds. Previous studies involving NPC proliferation on 2D alumina discs [32] yielded analogous results, with cells expanding all over the ceramic substrate. However, this current research extends those findings by successfully demonstrating NPC proliferation on 3D-printed scaffolds which can be fabricated by SLA printing technique [31,45]. This marks a significant advancement towards achieving controlled morphological characteristics of the ceramic substrate, essential for organoid or sensor applications, in which controlled shape and ceramic porous structure may facilitate vascularization, thereby providing the necessary nutrients and oxygen to neural tissues.

## Figures and Tables

**Table 1:** Cubic samples with calcination profile and wall thickness.

Sample	Calcination Profile	Wall Thickness (mm)
F-0.5	Fast	0.5
F-1	Fast	1
F-2	Fast	2
F-4	Fast	4
S-0.5	Slow	0.5
S-1	Slow	1
S-2	Slow	2
S-4	Slow	4

**Table 2:** Design-related parameters for the developed scaffolds.

Scaffolds	Channel Type	Channel Diameter (mm)	Porosity (%)
S1	Honeycomb	2	48.7%
S2	Circular	2	44.2%
S3	Honeycomb	1.5	27.4%
S4	Circular	1.5	44.2%
S5	Honeycomb	1	33.8%
S6	Circular	1	30.7%

**Table 3:** Main properties of 3D-printed silica parts along with the basic features of the 3D printing process.

Main Properties	
Print-out density	1.9 g/cm^3^
Elastic modulus	50 GPa
Poisson ratio	0.14
Shear modulus	21.9 Gpa
Yield strength	16.6 Mpa
Basic 3D printing features	
Layer height	50 μm
XY accuracy	25 μm
Printing time (per unit)	≈3 h
Material’s volume	1.07 mL

## Data Availability

No new data are created.

## Supplementary Materials

The following supporting information can be downloaded at: https://www.mdpi.com/article/10.3390/jfb14090465/s1, Figure S1: NPC-laden ceramic scaffolds by cell loading and by immersion in cell suspension (pore size: 2 mm, wall thick-ness: 0.5 mm) at day 4 and day 22. Table S1: Dimensions of the cubic samples with different wall thickness non calcined and under Slow and Fast calcination profiles and respective % reduction.
