# Supplementary material for: Fabrication and Optimization of 3D-Printed Silica Scaffolds for Neural Precursor Cell Cultivation
Source: J Funct Biomater. 2023 Sep 9;14(9):465. doi: 10.3390/jfb14090465 (PMC10531779; doi:10.3390/jfb14090465)
Supplement: Supplementary file 1 [file jfb-14-00465-s001.zip › jfb-2544715-supplementary.pdf]

Supplementary Material of

Article

Fabrication and Optimization of 3D-Printed Silica Scaffolds for Neural Precursor Cell Cultivation

Georgia Kastrinaki [1,*], Eleftheria-Maria Pechlivani [2,†], Ioannis Gkekas [3,†], Nikolaos Kladovasilakis [2], Evdokia Gkagkari [1], Spyros Petrakis [3] and Akrivi Asimakopoulou [1]

Cell loading

Immersion

in cell suspension

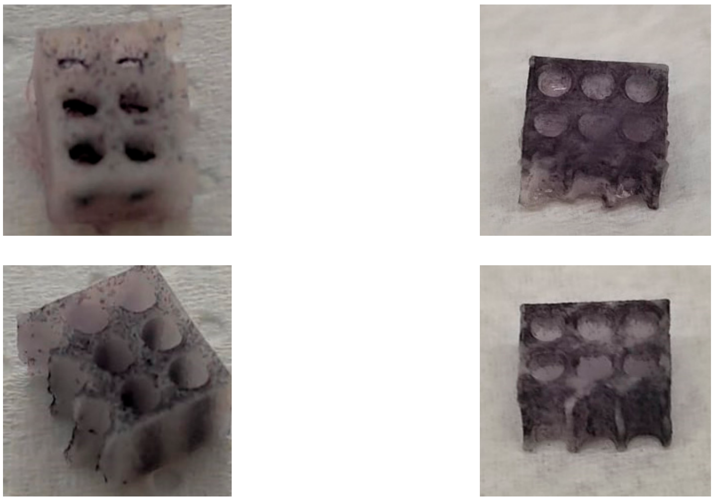

DAY 4 DAY 22

Figure S1. NPC-laden ceramic scaffolds by cell loading and by immersion in cell suspension (pore size: 2mm, wall thickness: 0.5mm) at day 4 and day 22

Wall thickness (mm)	Non calcined (mm)			Slow calcined (mm)			Fast calcined (mm)			Reduction of calcined					
										Slow calcined (%)			Fast calcined (%)		
-	x	y	z	x	y	z	x	y	z	x	y	z	x	y	z
0.5	22.8	22.3	28.6	19.3	18.6	-*	19.0	19.2	-*	15.2	16.1	-	16.50	13.54	
1	19.8	19.8	23.1	18.1	17.9	19.5	18.2	18.5	20.1	8.7	9.6	15.2	8.17	6.49	12.64
2	19.9	19.9	23.1	18.9	18.4	20.7	18.6	18.8	21.3	5.2	7.7	10.3	6.47	5.51	7.61
4	19.8	19.8	22.9	18.2	18.1	20.7	18.4	18.4	20.7	8.2	8.5	9.4	6.96	7.00	9.68

Table S1. Dimensions of the cubic samples with different wall thickness non calcined and under Slow and Fast calcination profiles and respective % reduction
